# Apollon modulates chemosensitivity in human esophageal squamous cell carcinoma

**DOI:** 10.18632/oncotarget.2293

**Published:** 2014-07-31

**Authors:** Si Zhang, Wenqing Tang, Shuqiang Weng, Xijun Liu, Benqiang Rao, Jianxin Gu, She Chen, Qun Wang, Xizhong Shen, Ruyi Xue, Ling Dong

**Affiliations:** ^1^ Key Laboratory of Glycoconjugate Research Ministry of Public Health, Department of Biochemistry and Molecular Biology, School of Basic Medical Sciences, Fudan University, Shanghai, China; ^2^ Department of Gastroenterology and Hepatology, Zhongshan Hospital, Shanghai Institute of Liver Disease, Fudan University, Shanghai, China; ^3^ Department of Gastrointestinal Anal Surgery and Institute of Gastroenterology, the Third Affiliated Hospital, Nanchang University, Nanchang, China; ^4^ Department of Thoracic Surgery, Zhongshan Hospital, Fudan University, Shanghai, China

**Keywords:** apoptosis, cancer, IAPs, prognosis, Smac

## Abstract

Patients with esophageal squamous cell carcinoma (ESCC) are often diagnosed with advanced diseases that respond poorly to chemotherapy. Here we reported that Apollon, a membrane-associated inhibitor of apoptosis protein, was overexpressed in ESCC cell lines and clinical ESCC tissues, and Apollon overexpression clinically correlated with poor response to chemotherapy (*P* = 0.001), and short overall survival (*P* = 0.021). Apollon knockdown increased cisplatin/docetaxel-induced apoptosis, mitochondrial dysfunction and cytochrome c release in two ESCC cell lines. Apollon knockdown potentiated cisplatin/docetaxel-induced long-term cell growth inhibition, and enhanced chemosensitivity of ESCC cells to cisplatin/docetaxel in xenograft tumor models. Apollon knockdown also enhanced cisplatin/docetaxel-induced activation of caspase-8 (extrinsic pathway) and caspase-9 (intrinsic pathway) in ESCC cells and xenograft tumor models. Mechanism studies revealed that the effect of Apollon on chemosensitivity is mainly mediated by Smac. Apollon expression strongly and negatively correlated with Smac expression in clinical ESCC tissues (*P* = 0.001). Apollon targeted Smac for degradation in ESCC cells. The effect of Apollon on chemosensitivity was reversed by Smac knockdown in ESCC cells. Taken together, our data show association of Apollon expression with chemotherapeutic response in ESCC, and provide a strong rationale for combining Apollon antagonism with chemotherapy to treat ESCC.

## INTRODUCTION

Esophageal cancer is the eighth most common incident cancer and the sixth most common cause of cancer-related death worldwide [1]. The presence of lymph node metastasis and vascular invasion as well as distant organ involvement leads to a poor clinical outcome and the 5-year overall survival (OS) rate is only 20% to 30% [2, 3]. Esophageal squamous cell carcinoma (ESCC), the major histological subtype of esophageal cancer, accounting for more than 90% of the total esophageal cancer occurrence globally, is often diagnosed with advanced diseases that respond poorly to chemotherapy and radiation therapy. Concurrent chemo/radiotherapy is currently regarded as the most effective treatment for advanced esophageal cancer. Cisplatin and docetaxel are used as monotherapy or in combination with other agents to treat ESCC clinically, but their activities are far from satisfactory [4, 5]. Therefore, identifying and targeting genes conducive to the treatment of ESCC, such as enhancement of conventional chemotherapy, is necessary to improve the survival of patients with this type of refractory cancer.

Induction of apoptosis is the main molecular mechanism of chemo- and radiotherapy to kill cancer cells [6]. Resistance to apoptosis is a common feature whereby tumor cells escape from chemotherapy-induced cytotoxicity, thus leading to chemoresistance. Apoptosis inhibitory genes with certain activity in human cancers function to promote carcinogenesis and tumor progression, including the ability to allow tumor cells to escape drug-induced apoptosis. The inhibitor of apoptosis proteins (IAPs) are crucial components of these processes because of their functional importance in the regulation of cell death [7]. IAPs play a role in apoptosis resistance in a variety of cancer cell lines and are characterized by a novel domain of 70 amino acids termed baculovirus inhibition of apoptosis protein repeat (BIR) [8, 9]. IAPs bind to and inhibit various pro-apoptotic factors, thereby effectively suppressing apoptosis induced by a broad range of effectors, including chemotherapeutics and irradiation. The aberrant high expression of IAPs in cancer cells, with little expression in most normal tissues, makes IAPs an attractive anticancer target [10].

Apollon, also called baculoviral IAP repeat containing 6 (BIRC6), is a unique member of the IAP family. It is a giant memberane-associated protein with 4830 amino acid residues with a N-terminal single BIR domain and a C-terminal ubiquitin-conjugating (UBC) enzyme domain [11]. Apollon can form a thioester with ubiquitin and transfer ubiquitin to substrate proteins, demonstrating that it functions as a chimeric E2/E3 ubiquitin ligase [12]. Ample evidence has indicated that Apollon is abnormally over-expressed in some kinds of malignant tumors including brain cancer, breast cancer, colon cancer, lung cancer and childhood acute leukemia [13-17]. Importantly, excessive expression of Apollon is significantly correlated with unfavorable clinical features at diagnosis, pathological grading of tumor, high cellular proliferation, and poor prognosis in acute myeloid leukemia and lung cancer [16-17].

Although the expression of several IAPs in ESCC and their role in chemoresistance have been previously investigated [18-21], the expression and role of Apollon in ESCC, however, remains unclear. In the present work, we studied the expression pattern of Apollon in ESCC cell lines and clinical ESCC tissues. In addition, we evaluated the association between Apollon expression and chemotherapy response in ESCC patients. We also investigated the molecular mechanisms by which Apollon regulates chemosensitivity in ESCC cells.

## RESULTS

### Apollon overexpression correlated with Smac downregulation in ESCC patients

**Fig.1:**
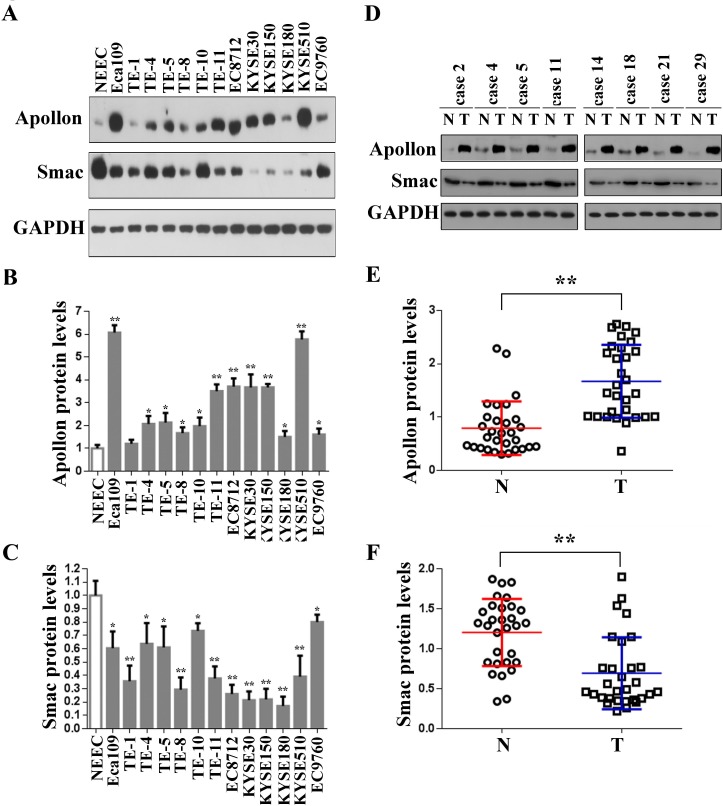
Overexpression of Apollon and downregulation of Smac in ESCC cell lines and clinical ESCC tissues (A) Protein levels of Apollon and Smac in normal human esophageal epithelial cells (NEEC) and cultured ESCC cell lines were analyzed by Western blotting. Apollon (B) and Smac (C) protein levels were quantificated in NEEC and cultured ESCC cell lines. Protein levels were normalized for GAPDH. Data are represented as Means ± standard deviations (SD) from three independent experiments. *P < 0.05 and **P < 0.01 vs. NEEC. (D) Typical patterns of Apollon expression analyzed by Western blotting in paired ESCC tissue samples. N, adjacent non-tumorous tissues; T, tumor tissues. Quantification of Apollon (E) and Smac (F) protein in 30 paired ESCC tissue samples. Protein levels were normalized for GAPDH. **P < 0.01.

We first determined the level of Apollon protein in 13 ESCC cell lines. Western blotting showed that Apollon protein was highly expressed in Eca109, TE-4, TE-5, TE-8, TE-10, TE-11, EC8712, KYSE30, KYSE150, KYSE180, KYSE510 and EC9760, whereas it was weakly detected in TE-1 and normal esophageal epithelial cells (NEECs) (Fig. 1A, upper panel and 1B). Recently, Hao *et al*. [22] reported that the second mitochondria-derived activator of caspase (Smac) was downregulated by Apollon in human 293T embryonic kidney cells. However, a contradictory conclusion was drawn by Ren *et al*. [23], who reported that the amount of Smac was not altered by Apollon in human H460 lung nonsmall carcinoma cell. Therefore the effect of Apollon on Smac appears to vary according to tissue types. To investigate whether Apollon regulates Smac in ESCC cells, we measured the level of Smac in 13 ESCC cell lines. In contrast to the increase of Apollon, Smac expression is decreased in all ESCC cell lines studied (Fig. 1A, middle panel and C). In addition, using Western blotting, we demonstrated the overexpression of Apollon (Fig. 1D, upper panel and 1E) and downregulation of Smac (Fig. 1D, middle panel and F) in the tumor tissues from 30 patients with ESCC. Moreover, Apollon expression negatively correlated with Smac expression (R = -0.463, *P* = 0.009) (Supplementary Figure 1).

**Fig.2:**
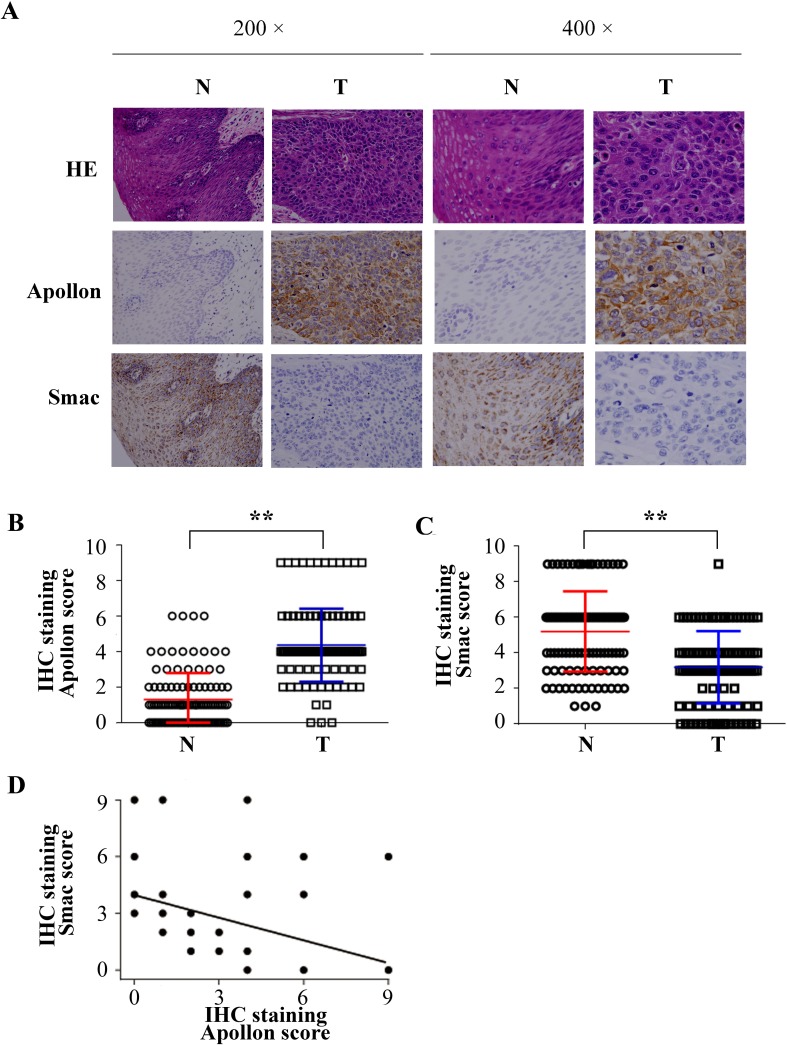
Negative correlation between Apollon expression and Smac expression in clinical ESCC samples (A) Typical patterns of Apollon and Smac staining in paired ESCC tissue samples. N, adjacent non-tumorous tissues; T, tumor tissues. Scores of immunochemistry staining of Apollon (B) and Smac (C) in 111 ESCC patients. **P < 0.01. (D) Correlations between Apollon scores and Smac scores in tumor tissues of 111 ESCC patients.

To further evaluate the role of Apollon in human ESCC, we next examined Apollon expression in tissues from 111 patients with ESCC using immunohistochemistry (IHC) staining. Positive signals of Apollon mainly localized in the cytoplasm. High staining of Apollon could be observed in 62 of 111 (55.8%) cases of ESCCs, whereas in only 12 of 111 (10.8%) cases of adjacent non-tumor tissues. The Apollon scores in tumor tissues were 2.4-fold higher than those in adjacent non-tumor tissues (Fig. 2A and B). Considering the possible relationship between Apollon and Smac, Smac was also stained in the same series of patients. In contrast to Apollon, the Smac scores were 1.8-fold lower in ESCC tumor tissues than in adjacent non-tumor tissues (Fig. 2A and C). Notably, Apollon expression strongly and negatively correlated with Smac expression (R = -0.416, *P* = 0.001) (Fig. 2D). We further investigated the correlation of Apollon expression with clinicopathologic features. Clinical characteristics of patients are listed in Supplementary Table 1. Clinicopathologic analysis showed that Apollon expression failed to correlate to the clinical pathological factors including TNM stage and tumor differentiation (Supplementary Table 2). To give a comprehensive evaluation of IAPs expression in human ESCC, we detected other members of IAPs by IHC staining in 111 patients with ESCC. We found that c-IAP1 (Birc2), XIAP (Birc4), Survivin (Birc5) and Livin (Birc7) were overexpressed in ESCC tissues, while NAIP (Birc1) and c-IAP2 (Birc3) were comparable between tumor tissues and adjacent non-tumor tissues (Supplementary Fig. 2).

### Apollon expression correlated with the chemotherapeutic response in ESCC patients

**Fig.3:**
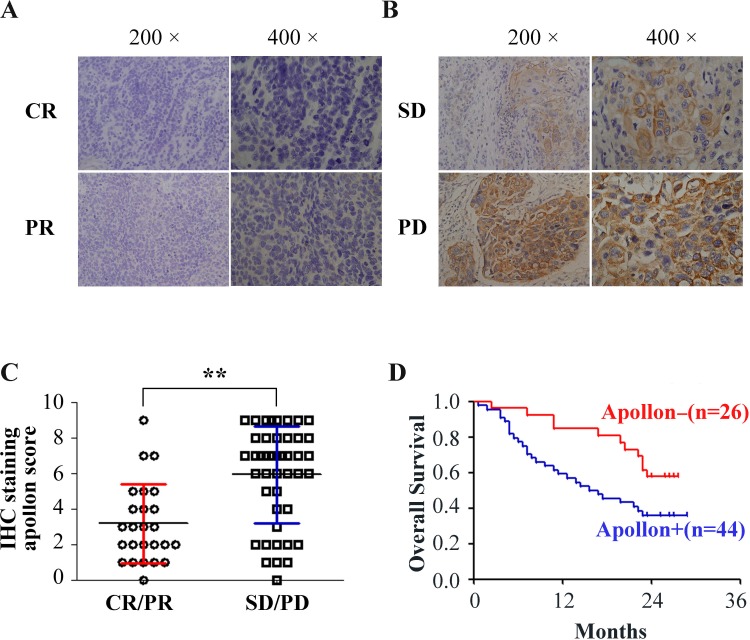
The association of Apollon expression with the chemotherapeutic response and survival outcome in ESCC patients undergone cisplatin-based chemotherapy (A-B) Typical patterns of Apollon staining in tumor tissues of 70 ESCC patients with differential chemotherapeutic response. The esophageal cancers with complete response (CR)/partial response (PR) (A) responses to cisplatin-based chemotherapy expressed lower levels, whereas those with stable disease (SD)/progressive disease (PD) (B) responses expressed higher levels of Apollon. (C) Scores of immunochemistry staining of Apollon in 70 ESCC patients with different chemotherapeutic response. **P < 0.01. (D) Kaplan-Meier analysis of overall survival (OS) for Apollon expression in 70 ESCC patients undergone cisplatin-based chemotherapy.

To study whether there is a relationship between Apollon expression and chemotherapeutic response in ESCC patients, we analyzed another cohort of 70 ESCC patients who had undergone cisplatin-based chemotherapy. Clinical characteristics of patients are listed in Supplementary Table 3. With regard to the clinical response, chemotherapy-sensitive with complete response (CR)/partial response (PR) was achieved in 25 patients, whereas chemotherapy-resistant with stable disease (SD)/progressive disease (PD) was observed in 45 patients. High staining of Apollon in tumor tissues could be observed in 9 (36%) patients of CR/PR group, but in 35 (77.8%) of SD/PD group. Interestingly, we found that the expression of Apollon in tumor tissues inversely and significantly correlated with the clinical response to chemotherapy (*P* = 0.001) (Fig. 3A, B and Table 1). Apollon expression in tumor tissues of SD/PD groups was 1.9-fold as high as that in CR/PR groups (Fig. 3C). However, Apollon did not obviously correlate with other clinical and pathologic characteristics, including TNM stage and tumor differentiation (Table 1). Notably, Kaplan-Meieranalysis showed that overall survival (OS) was significantly worse among patients with Apollon-staining high (*P* = 0.012) (Fig. 3D).

**Table 1:** Correlation of chemotherapeutic response and clinicopathologic features with Apollon expression

	expression of Apollon		*P* values	OR (95%CI)
	Negative	Positive		
Patients	26	44		
Age, years				
≤56	8	14		
>56	18	30	0.927	0.952 (0.334-2.713)
Lymph nodes metastasis				
No	11	21		
Yes	15	23	0.660	0.803 (0.302-2.134)
Distant metastasis				
No	6	9		
Yes	20	35	0.796	1.167 (0.362-3.759)
Invasive depth				
Mucosa to muscularisproria	10	14		
Adventitia to adjacent structure	16	30	0.572	1.339 (0.486-3.689)
Tumor size (cm)				
≤ 4	12	15		
> 4	14	29	0.318	1.657 (0.615-4.466)
Position				
Upper and middle	21	37		
Hypomere	5	7	0.722	0.795 (0.224-2.819)
TNM stage				
I, II	15	17		
III, IV	11	27	0.125	2.166 (0.807-5.809)
Tumor differentiation				
I, II	18	27		
III	8	17	0.508	1.417 (0.506-3.970)
Chemosensitivity				
CR + PR	16	9		
SD + PD	10	35	**0.001**	6.222 (2.119-18.275)

Bold *P* value less than 0.05 indicates statistical significance.

### Apollon knockdown potentiated cisplatin and docetaxel induced apoptosis in ESCC cells

**Fig.4:**
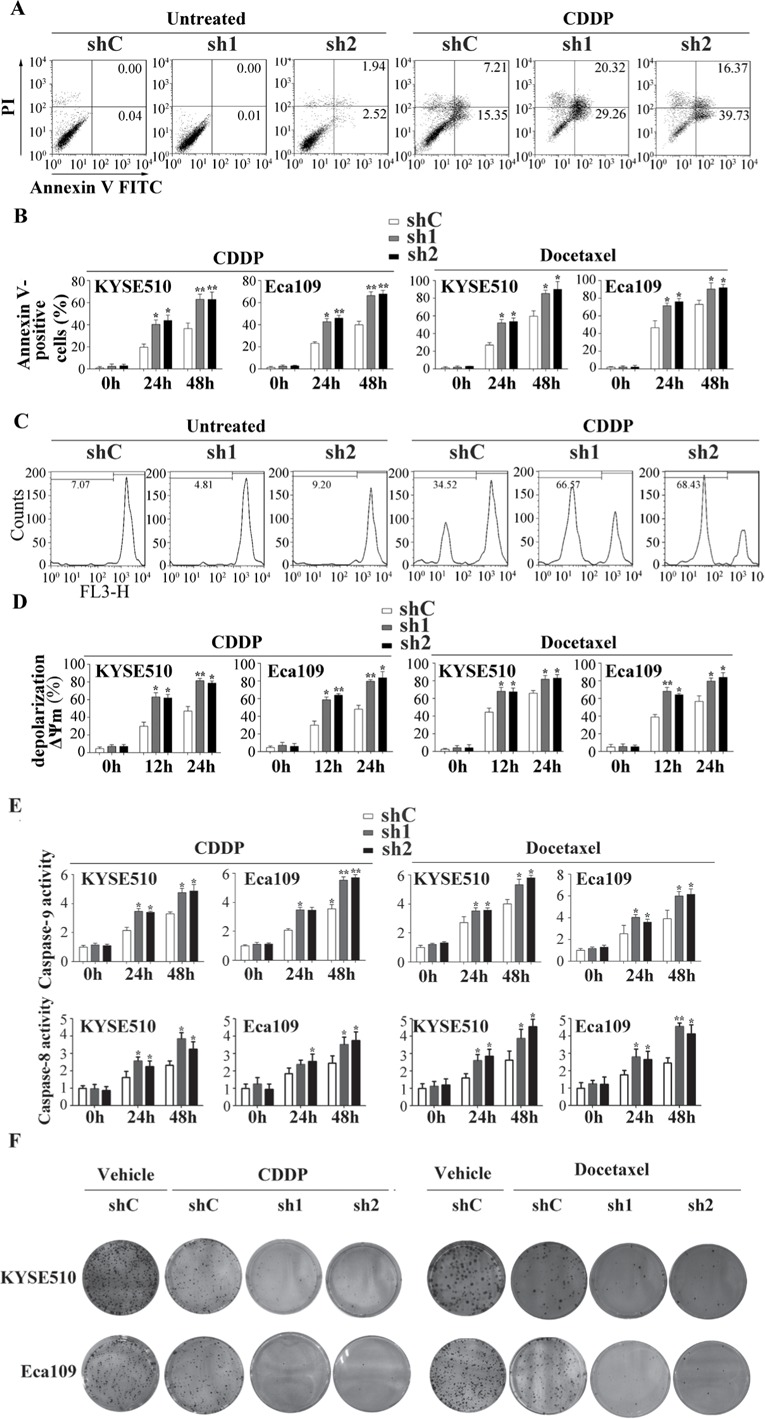
Effect of Apollon knockdown on the chemosensitivity of ESCC cells to cisplatin (CDDP) and docetaxel *in vitro* (A) KYSE510 cells transfected with control shRNA (shC) or two different shRNAs against human Apollon (sh1, sh2) were treated with CDDP (10 μM) for 24 hours, then apoptosis was detected by Annexin V–PI staining. (B) KYSE510 cells and Eca109 cells transfected with control shRNA (shC) or Apollon shRNAs (sh1, sh2) were treated with CDDP (10 μM) or docetaxel (10 nM) for the indicated times. Apoptosis was quantified by annexin V-positive cells. (C) KYSE510 cells transfected with control shRNA (shC) or Apollon shRNAs (sh1, sh2) were treated with CDDP (10 μM) for 24 hours, then Mitochondrial inner transmembrane potential (Δψm) depolarization was detected by Mito Tracker Red staining. (D, E) KYSE510 cells and Eca109 cells transfected with control shRNA (shC) or Apollon shRNAs (sh1, sh2) were treated with CDDP (10 μM) or docetaxel (10 nM) for the indicated times, Δψm depolarization was quantified as the percentage of depolarized cells. Caspase-9 and caspase-8 activation were quantified using a luminescent reporter. All data are represented as Means ± standard deviations (SD) from three to four independent experiments. *P < 0.05 and **P < 0.01 vs. shC. (F) KYSE510 cells and Eca109 cells transfected with control shRNA (shC) or Apollon shRNAs (sh1, sh2) were treated with vehicle, CDDP (10 μM) or docetaxel (10 nM) for 6 hours, long-term cell viability was assessed by the colony formation assay. Representative pictures are shown.

Cisplatin (known as cis-diammine-dichloroplatinum II) and docetaxel (known as Taxol®, a semi-synthetic analogue of paclitaxel) are clinically used in adjuvant or neoadjuvant chemotherapy for ESCC. Resistance to cisplatin/docetaxel remains a major problem in the treatment of ESCC patients. To investigate the role of Apollon in chmosensitivity of ESCC cells to cisplatin/docetaxel, we used shRNA to knock down Apollon expression in KYSE510 and Eca109 cells. The effect of Apollon knockdown was confirmed with Western blotting (Supplementary Fig. 3). Apollon knockdown significantly increased cisplatin- or docetaxel-induced apoptosis at different time points, as determined by Annexin V/PI staining (Fig. 4A and B). Mitochondrial membrane potential (ΔΨm) depolarization is an early apoptosis indicator reflecting mitochondrial disruption. Apollon knockdown strongly potentiated cisplatin- or docetaxel-induced ΔΨm depolarization at different time points, as detected by Mito Tracker Red staining (Fig. 4C and D). Interestingly, Apollon knockdown also enhanced cisplatin- or docetaxel-induced caspase-9 and caspase-8 activation, as measured by caspase activity assay (Fig. 4E). Moreover, Apollon knockdown enhanced cisplatin- or docetaxel-induced long-term cell growth inhibition, as shown by long-term cell viability with colony formation assay (Fig. 4F and Supplementary Fig. 4).

### Apollon modulated chemosensitivity via Smac in ESCC cells

**Fig.5:**
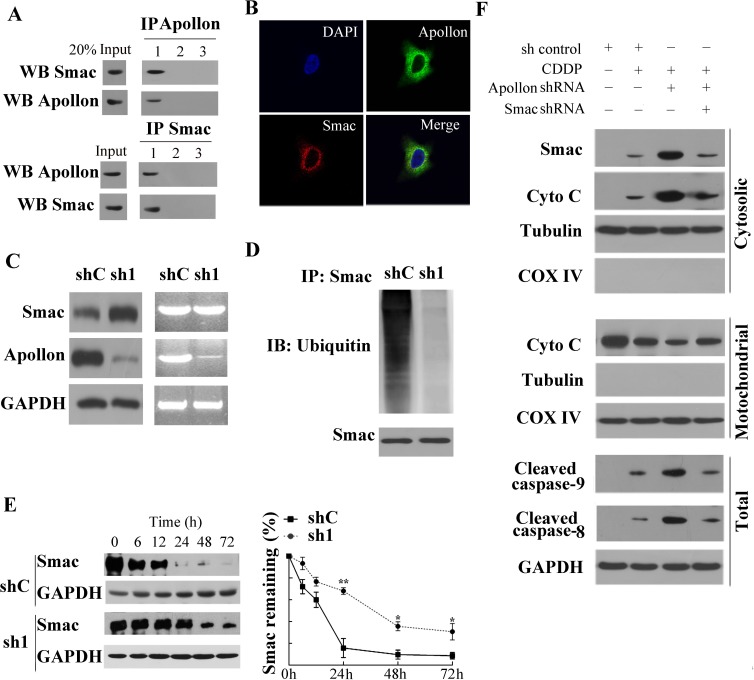
Apollon knockdown enhanced the chemosensitivity of ESCC cells by increasing Smac stability (A) Human ESCC tissue lysates were co-immunoprecipitated with: lane 1, anti-Apollon/anti-Smac antibody; lane 2, isotype control antibody; lane 3, PBS and immunoblotted with the indicated antibody. (B) Double immunofluorescence staining in KYSE510 cells: the KYSE510 cells were incubated with specific antibodies against Apollon (green) and Smac (red) respectively, merged images showed protein co-localization. (C) The translation level and transcription level of Smac in KYSE510 cells transfected with control shRNA (shC) and Apollon shRNA (sh1). (D) The level of ubiquitinated Smac in KYSE510 cells transfected with control shRNA (shC) or Apollon shRNA (sh1). Cells were treated with MG132 (20 μΜ, 12 h) before harvest for protein detection. (E) Apollon knockdown increased Smac stability in KYSE510 cells. Cells were treated with cycloheximide (20 μM, 12h), and harvested for protein detection at indicated time points. Left panel: immunoblots of Smac. Right panel: Relative remaining of Smac. Data are represented as Means ± standard deviations (SD) from three independent experiments. *P < 0.05 and **P < 0.01 vs. shC. (F) Smac knockdown reversed the potentiated effect of Apollon knockdown on cisplatin (10 μM) induced Smac and cytochrome c release, caspase-9 and caspase-8 activation. Mitochondrial and cytosolic fractions were isolated from the treated cells and analyzed for the indicated proteins with Western blotting.

The observation that Apollon expression strongly and negatively correlated with Smac expression in ESCC tissues attracted our attention, and provided motivation to study the effects of Apollon on Smac in ESCC tissues and cells. Using co-immunoprecipitation (co-IP) assays, we found that Apollon bound to Smac in human ESCC tissue (Fig. 5A). To verify the stringency of co-IP analysis, parallel experiments were conducted in which the co-IP antibody was replaced with an isotype-matched control antibody or PBS. Moreover, we observed the interaction between Apollon and Smac in KYSE510 cells (data not shown). We next determined the localization of these two proteins in KYSE510 cells with immunofluorescence analysis. Co-localization of Apollon and Smac in cytoplasm was shown by overlapping fluorescent signals (Fig. 5B). To investigate the effect of Apollon on Smac, we examined the Smac levels in Apollon knockdown KYSE510 cells. We found that Smac protein level was dramatically upregulated, whereas Smac mRNA level was not affected, by Apollon knockdown (Fig. 5C). In contrast, the level of ubiquitinated Smac was reduced by Apollon knockdown in KYSE510 cells (Fig. 5D). Smac has been reported to be degraded by ubiquitin-proteasomal pathway. Therefore, we measured Smac degradation in Apollon knockdown KYSE510 cells. Cells were treated with a protein synthesis inhibitor cycloheximide for the indicated period of time before harvesting. Levels of Smac were markedly decreased in control cells but remained relatively high in Apollon knockdown cells over time (Fig. 5E).

To better understand the mechanism underlying Apollon knockdown-mediated enhancement of chemosensitivity, we performed Western blotting to investigate apoptotic signaling. Our data showed that Apollon knockdown potentiated cisplatin-induced Smac release, along with cytochrome c release, caspase-9 and caspase-8 activation in KYSE510 cells. These effects were reversed by shRNA-mediated Smac knockdown (Fig. 5F). Consistent with Western blotting analysis, Annexin V/PI staining and Mito Tracker Red staining demonstrated that Apollon knockdown potentiated cisplatin- or docetaxel-induced apoptotic events, which were reversed by Smac knockdown (Supplementary Fig. 5A and B). Consistently, Smac knockdown reversed the facilitated effects of Apollon knockdown on cisplatin- or docetaxel-induced long-term cell growth suppression in KYSE510 and Eca109 cells (Supplementary Fig. 5C)

### Apollon modulated chemosensitivity to cisplatin and docetaxel *in vivo*

**Fig.6:**
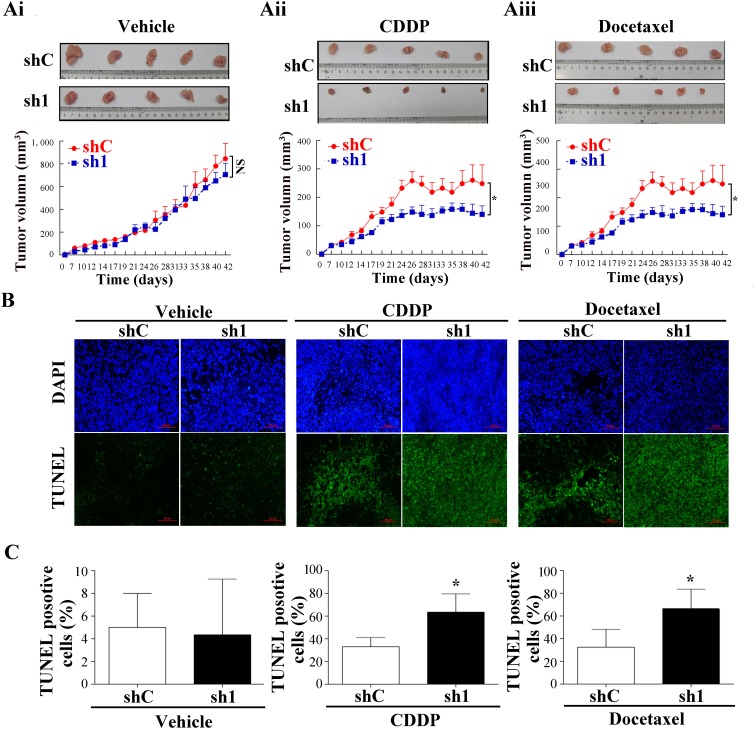
Effect of Apollon knockdown on the chemosensitivity of ESCC cells in xenograft tumor models KYSE510 cells stably transfected with control shRNA (shC) and Apollon knockdown shRNA (sh1) were injected subcutaneously into nude mice. One week after inoculation, tumorbearing mice were treated with cisplatin or docetaxel. (Ai-Aiii) The tumor size was monitored and calculated as the mean tumor volume ± standard deviations (SD) of 5 mice of each group. Upper pannel: Representative images of harvested tumors of each group. Lower panel: *In vivo* subcutaneous tumor growth curves. *P < 0.05. (B) Representative TUNEL staining in tumors of each group. (C) Quantification of TUNEL positive cells in tumors of each group. (D) Representative immunohistochemistry staining for cleaved caspase-9 and cleaved caspase-8 in tumors of each group. (E) Quantification of cleaved caspase-9 positive cells and cleaved caspase-8 positive cells in tumors of each group. Data are represented as the Mean ± SD of each group. *P < 0.05 and **P < 0.01 vs. shC. (F) Representative immunohistochemistry staining for Apollon and Smac in tumors of each group.

To determine whether Apollon knockdown increases chemosensitivity *in vivo*, shC and sh1 KYSE510 cells were injected subcutaneously into flanks of nude mice. After the tumor volume reached 50 to 90 mm^3^, tumorbearing mice were treated with cisplatin (4 mg/kg; i.p., twice weekly) or docetaxel (7.0 mg/kg; i.p., thrice weekly) as described previously with modifications [24, 25]. The antitumor efficacy was measured by monitoring the tumor volume after treatment. The average tumor volume was slightly decreased in Apollon knockdown cells compared to control cells, but the difference did not reach statistical significance (Fig. 6Ai). Following the treatment with cisplatin (docetaxel), the average tumor volume of control cells and Apollon knockdown cells was 400.2 ± 20.6 mm^3^ (248.2 ± 26.69 mm^3^) and 219.7 ± 16.6 mm^3^ (129.2 ± 12.5 mm^3^), respectively (Fig. 6Aii and Aiii). Apollon knockdown resulted in 45.1% and 47.9% inhibition of tumor growth in cisplatin and docetaxel-treated mice, respectively. Apollon knockdown enhanced cisplatin- or doxetacel- induced apoptosis in tumor cells *in vivo*, as shown by TUNEL assay (Fig. 6B and C). Apollon knockdown also increased cisplatin- or doxetacel- induced caspase-9 and caspase-8 activation *in vivo*, as assessed by IHC staining (Fig. 6D and E). Finally, Apollon knockdown increased Smac expression *i*n *vivo* (Fig. 6F).

## DISCUSSION

This study was done to investigate the possible role of Apollon in ESCC chemosensitivity for the first time. Western blotting and immunohistochemistry demonstrated Apollon overexpression in ESCC cell lines and clinical ESCC tissues. Although we failed to find any correlation between Apollon expression and stage or grade for ESCC, we did find that high expression of Apollon was closely associated with poor response to chemotherapy in patients who had undergone cisplatin-based chemotherapy. Clinical pathological factors including TNM stage and tumor differentiation usually have great impact in deterring the response to chemotherapy. Meanwhile, clinical pathological factors may not be the only elements that influence sensitivity to chemotherapy. Abnormal protein expression and disordered signaling pathway, which impede apoptosis, can also lead to the resistance to chemotherapy. Recently, an attempt has been made to identify the genomic alterations in human ESCC, which reveals that somatic aberrations are mainly involved in cell survival and apoptosis pathways [26]. In the present study, we found that Apollon knockdown alone is unable to alter tumor growth in xenograft tumor model, which was consistent with our finding that Apollon expression did not correlate with the stage and grade of ESCC. Nevertheless, Apollon, as a unique member of IAPs, altered the apoptotic threshold of ESCC cells *in vitro* and *in vivo* in our study. Moreover, high expression of Apollon was associated with short overall survival in patients undergone cisplatin-based chemotherapy. These data suggest a previously unrecognized role of Apollon in the intrinsic resistance of esophageal cancer to chemotherapy.

RNA interference has become conventional applications for *in vivo* cancer therapy, and an efficient way of delivering small interfering RNA into solid tumors has been developed [27]. Using RNA interference, we found that Apollon knockdown significantly potentiated cisplatin or doxetacel-induced apoptosis, mitochondrial membrane potential collapse, cytochrome c release, caspase activation, and long-term cell growth suppression, hence sensitizing esophageal cancer to cisplatin or doxetacel treatment *in vitro* and *in vivo*. This critical role of Apollon in chemotherapy-induced apoptosis is also supported by previous studies on Apollon using other agents and different cell types [28, 29]. Apoptosis is one of the main contributors, although certainly not the only one, to the cytotoxic effect of chemotherapeutic drugs. Indeed, proteins that control the apoptotic process hold great promise as drug targets for various tumor types. In the present study, aberrant Apollon expression in ESCC may confer chemoresistance by altering the balance between pro- and anti-apoptotic proteins. Apollon knockdown may have the potential to restore chemosensitivity in resistant cells.

We further observed that Smac appeared to be dispensable for the activity of Apollon on chemosensitivity in ESCC cells. So far, the association between Apollon and Smac is not well understood and inconsistent results have been reported. Hao *et al*. [22] reported that Apollon facilitated Smac degradation in human 293T embryonic kidney cells *in vitro*, whereas Ren *et al*. [23] reported that Apollon did not promote Smac degradation in human H460 lung nonsmall carcinoma cell *in vitro*. Both Hao and Ren [22, 23] reported that Apollon deletion did not affect Smac protein levels in Apollon deficient mice *in vivo*. However, Qiu *et al*. [30] reported that Apollon affect Smac protein levels in wild-type mice tissues *in vivo*. Other researchers [31, 32] reported that Apollon may also regulate Smac mRNA levels. Based on these results, we proposed that effect of Apollon on Smac may vary according to species and tissue types. In the present studies, we found that Apollon was overexpressed, while Smac was downregulated, in ESCC cells and tissues. Apollon overexpression strongly correlated with Smac downregulation in clinical ESCC tissues. Apollon knockdown increased Smac protein level, while had no effect on Smac mRNA level in ESCC cells. Apollon interacted with Smac in clinical ESCC tissues. Moreover, Apollon ubiquitinated Smac, consequently facilitated its proteasomal degradation in ESCC cells. Most importantly, Smac knockdown reversed the sensitization effect of Apollon knockdown on ESCC cells to chemotherapy. Taken together, these data underscore an important role of Smac in Apollon mediated chemoresistance. This role was supported by previous studies by Xu *et al*., who reported that downregulation of Smac may be a chemoresistance mechanism in ESCC cells [25].

Smac [known as DIABLO (direct IAP binding protein with low pI) in mouse], is a recently identified mitochondrial protein. Upon chemo treatment, Smac is released along with cytochrome c and promotes cytochrome c-dependent caspase activation by neutralizing inhibitor of apoptosis proteins. A growing number of studies have reported the association between IAPs and Smac. Cytoprotective IAPs, ML-IAP (melanoma IAP) and ILP-2 (IAP-like protein 2), exert their antiapoptotic effects through the neutralization of Smac [33, 34]. C-IAP1 and c-IAP2 stimulate Smac degradation as E3 ubiquitin ligases both *in vivo and in vitro* [35]. XIAP interacts and retains Smac inside mitochondria that already underwent MOMP (mitochondrial outer membrane permeabilization) [36]. In our studies, apollon appears to play an important role in cytoprotection against Smac, which contributes to the chemoresistance in ESCC.

A surprising finding was that apollon knockdown enhanced activation of both caspase 9 and caspase 8 in cisplatin/docitaxel treated ESCC cells *in vitro* and *in vivo*. Caspase 9 and caspase 8 have been recognized as the initiator caspases of intrinsic and extrinsic apoptosis pathways, respectively [37]. We, hence, proposed that Apollon may regulate chemosensitivity via multifaceted mechanism involving both intrinsic mitochondrial and extrinsic death receptor pathway. Although Apollon and Smac typically regulated apoptosis through the mitochondrial intrinsic pathway [14, 23, 25], recently Probst *et al*. [38] reported that Smac mimetics increased cancer cell response to chemotherapeutics through a TNF-a/RIP1-dependent extrinsic apoptotic pathway. We proposed that Apollon knockdown may promote cytochrome c-dependent caspase-9 activation and TNF-a/RIP1-dependent caspase-8 activation through Smac stabilization. Nonetheless, the underlying mechanism regarding the role of Apollon in intrinsic and extrinsic apoptosis pathways may need further investigation.

IAP antagonists have attracted increasing interest in triggering cancer cell death and are currently at the preclinical development stage or early clinical testing [39]. So far, six molecules targeting IAPs, including LCL-161, AT-406, HSG1029, TL32711, GDC-0152, CUDC-427, have entered clinical trials with early results showing no dose limiting toxicities, suggesting that IAP proteins can be targeted by small molecules [40]. Although a specific peptide or peptidomimetic against Apollon has not yet been developed, studies on protein–protein interaction domain and phase display have successfully developed inhibitors against XIAP, c-IAP1 and c-IAP2 [39]. Similar methods can be used to develop a specific antagonist of Apollon.

The present study has certain limitations. First, the clinical results were based on retrospective analysis by using tissue specimens obtained from patients who had undergone cisplatin-based chemotherapy at only one institution. Second, the current results showed that Apollon mainly modulated the chemosensitivity in esophageal cancer through regulation of Smac. However, we cannot exclude the possibility that Smac is an important but not exclusive mediator of the chemoresistant effects of Apollon, because Apollon was reported to function as an E2/E3 ubiquitin ligase to ubiquitinate other apoptosis effector [12, 14, 22, 23]. Third, the present study was carried out in certain cell lines. Considering that most cancer tumors are highly heterogeneous, we need to validate our findings in other ESCC cell lines.

In conclusion, our results show association of Apollon overexpression with chemotherapeutic response. These results suggest that combinations of conventional anticancer drugs with Apollon-targeted therapy may be beneficial for the treatment of esophageal cancer. Further studies of Apollon antagonist and other apoptosis regulators may help develop novel therapeutic strategies for esophageal cancer.

## MATERIALS AND METHODS

### Patients' samples

For Apollon and Smac expression studies, Tumor specimens were obtained from 111 consecutive male patients with ESCC who have undergone curative resection in Zhongshan Hospital, Fudan University between 2007 and 2008. Patient clinical parameters were summarized in Supplementary Table 1. The inclusion and exclusion criteria have been reported [41]. Briefly: (a) having a distinctive pathologic diagnosis of ESCC, (b) having no anticancer treatment before esophageal resection, (c) having curative esophageal resection, and (d) having suitable formalin-fixed and paraffin-embedded tissues. Curative resection was defined as complete resection of all tumor nodules and the cut surface being free of cancer by histologic examination. Each specimen has a corresponding nontumorous tissue 3 cm away from the tumor. ESCC diagnosis was based on the World Health Organization (WHO) criteria. Tumor differentiation and staging was defined according to the 7th edition of tumor-node-metastasis (TNM) classification of Union Internationale Contra Cancrum (UICC).

For 70 response cases of chemotherapy (70 male), included CR/PR and SD/PD, tumor specimens were obtained from the third affiliated hospital, Nanchang University between January, 2005 and December, 2008. Patient clinical parameters were summarized in Supplementary Table 2. All patients were diagnosed as ESCC histopathologically and subjected to surgical esophagectomy, followed by cisplatin-based chemotherapy for postoperative recurrence. Cisplatin was given by continuous intravenous administration at a dose of 60 to 100 mg/m^2^ for 4 to 5 days, and in combination of paclitaxel, 5-FU, or gemcitabine [42]. The effect of chemotherapeutic response was evaluated clinically according to WHO criteria. The diagnostic examinations consisted of esophagography, computed tomography, chest X-ray, abdominal ultrasonography, and bone scan. The subjects were followed-up every 3-4 months during the first postoperative year and at least 6 months afterward for survival and recurrence inquiry until death, contact failure, or until the end of the investigation, i.e., January 10, 2012, with the median follow-up was 30 months (range 1–48 months).

### Antibodies

Primary antibodies against Survivin (#2808), Apollon (#8745), Livin (#5471), Smac (#2954), cleaved caspase-8 (#9496), cleaved caspase-9 (#9505), cytochrome c (#4272), α-tubulin (#2144), COX IV (#4844), ubiquitin (P4D1, #3936) were purchased from Cell Signaling Technology (Beverly, MA). Antibodies against glyceraldehyde-3-phosphate dehydrogenase (GAPDH) (#sc-47724) were from Santa Cruz Biotechnology (Santa Cruz, CA). Antibodies against NAIP (#ab25968), XIAP (#ab28151), c-IAP1 (#ab108361), c-IAP2 (#ab137393) were from Abcam (Cambridge, MA). Secondary antibodies for immunofluorescence were TRITC-conjugated donkey anti-mouse IgG and Alexafluor 488–conjugated goat anti-rabbit IgG, from Jackson ImmunoResearch Laboratories (West Grove, PA).

### Cell lines, culture conditions

Primary cultures of normal esophageal epithelial cells (NEEC) were established from fresh specimens of the adjacent noncancerous esophageal tissue, which is over 5 cm from the cancerous tissue, according to previous report [43]. Human ESCC cell lines Eca109, TE-1, TE-10, TE-11, EC8712 and EC9706 were obtained from Cell Bank of Type Culture Collection of Chinese Academy of Sciences (Shanghai, China). TE-4, TE-5, TE-8 were obtained from RIKEN Bioresource Center. Kyse30, Kyse150, Kyse180, Kyse510 were obtained from DSMZ, the German Resource Centre for Biological Material [44]. Cell lines were routinely maintained in RPMI 1640 or DMEM medium plus 10% fetal calf serum at 37°C in a humidified incubator under 5% CO_2_. All cell lines were authenticated by short tandem repeat fingerprinting on 10/2013.

### Lentiviral particles and transduction

The lentiviral particles obtained by short-hairpin RNA constructs targeting Apollon were purchased from the TRC-Mm1.0 library (Sigma Aldrich). The target set used for Apollon (NM_016252) included TRCN0000004157, TRCN0000004158, TRCN00000059, TRCN0000004160, and TRCN0000004161. The pLKO.1-puro lentiviral vector containing a sequence targeting luciferase, TGACCAGGCATTCACAGAAAT was used as the control (shC). KYSE510 cells and Eca109 cells were transfected with lentiviral particles, screened by puromycin (1 μg/ml) and then testified by Western blotting. Of these constructs, transduction of cells with TRCN0000004158 (sh1) and TRCN00000059 (sh2) produced the best knockdown. Knockdown of Smac was achieved by the expression of shRNAs targeting the sequence GUCUUAUUUACGUCGUCAA in Smac (shSmac) as reported previous [45].

### Immunohistochemistry

Paraffin-embedded sections (5 μm) were deparaffinized in xylene and rehydrated in a decreasing ethanol series diluted in distilled water. Following antigen retrieval with a 10 mM citrate buffer, ESCC slides were incubated overnight at 4°C with primary antibody. Afterwards the specimens were incubated with a peroxidase labeled polymer conjugated to secondary antibody for 30 min. They were washed again, and 3, 3′-diaminobenzidine (DAB) was used as a chromogen to visualize the reaction. Negative controls were obtained by substituting primary antibodies with non-immune serum.

The evaluation of immunohistochemical staining was carried out by two independent pathologists who were unaware of the patient outcomes. The ESCC slides were observed under a light microscopy, for a histological review in each case, to examine tumor microheterogeneity in antigen distribution. Five randomized microscopic views of 200-fold magnification of each slice were observed and scored. A semiquantitative scoring system was used that was based on both the staining intensity (0, negative; 1, weak; 2, intermediate; 3, strong) and the percentage of positive cells (0, 0% positive cells; 1, 1-10%positive cells; 2, 11-50% positive cells; 3, >50% positive cells). The final score of each sample was obtained by multiplying the scores for staining intensity and percentage of cells. Samples were classified as negative when the final scores were 0 to 3 and positive when 4 to 9.

### Apoptosis assays

For detection of mitochondrial membrane potential change, cells were stained by Mito Tracker Red CMXRos (Molecular Probes, Eugene, OR) for 15 min at 37°C and analyzed by flow cytometry, according to the manufacturer's instructions. For colony formation assays, cells were treated with drugs, and then seeded in 6-cm dishes (10^5^ cells per dish) and cultured in DMEM supplemented with 10% FBS without anticancer drugs. After 14 to 16 days, cells were fixed in 3.7% formaldehyde and stained with 0.25% crystal violet (Amresco, Solon, Ohio) in PBS for 30 minutes. Clones were imaged using Nikon ECLIPSE TE300 and macroscopically visible clones in 3 randomly chosen fields per well were counted for quantification [25]. Caspase-9 and caspase-8 activity was determined using the caspase-9 activity assay kit (R&D Systems Inc, Minneapolis, MN) and Caspase-Glo 8 assay kit (Promega, Madison, WI), according to the manufacturer's instructions, respectively. The specific caspase activity was expressed as a fold of the baseline caspase activity of the control sample. Each experiment was repeated at least three times. TUNEL staining in tissue sections was performed by using in situ cell death detection kit (Roche Applied Science, Indianapolis, IN, USA) according to manufacturer's instructions. Detection and quantification of cleaved caspase in tissue sections were carried out as reported previous [46].

### Subcellular fractionation and Western blotting

The mitochondrial and cytosolic fraction of cells was isolated using a mitochondria isolation kit (Pierce, Rockford, IL, USA) according to the manufacturer's instructions. Western blotting was carried out as described previously [25]. Each experiment was repeated at least three times.

### Co-IP and Immunofluorescence

Co-IP was carried out as described previously [47]. In brief, the ESCC tissue lysates were precleared with protein A-Sepharose beads (Roche, Indianapolis, IN) at 4°C for 8 h, and then incubated with protein A-agarose beads and antibodies at 4°C overnight. The precipitates were pelleted and washed three times with the lysis buffer before elution in SDS-loading dye and Western blotting. Immunofluorescence was carried out as described previously [47]. In brief, KYSE510 cells were fixed in ice-cold methanol for 1 h and blocked in PBS containing 10% normal blocking serum followed by overnight incubation at 4°C with the primary antibodies: monoclonal mouse anti-Apollon antibody and monoclonal rabbit anti-Smac antibody. After overnight incubation, the coverslips were rinsed three times in PBS and incubated at 37°C for 1 h with fluorescein isothiocyanate (FITC)-conjugated anti-mouse IgG and rhodamine-conjugated anti-rabbit IgG. The slides were then stained with DAPI, washed with PBST, mounted, and analyzed by confocal laser scanning microscopy (Leica TCS, Germany).

### Tumor xenograft models

Thirty male BALB/C nude mice (4 weeks of age, 12–14 g) were purchased from Slac Laboratory Animal Co. Ltd., Chinese Academy of Sciences (Shanghai, China) and were raised under specific pathogen-free conditions. All surgery was performed under anesthesia with sodium pentobarbital. To assess the tumourigenicity of BIRC6-knockdown stable clones, 10^7^ cells of KYSE510 cells (stable Apollon sh1 and the control clones) in 0.1 ml of PBS were injected subcutaneously into the right flank of each mouse. Tumor sizes were recorded thrice a week. Mice were sacrificed at 6 weeks post-injection; tumors were excised. Tumor volume was calculated by the formula: 0.5 ×L × W^2^ (L = length of tumor; W = width of tumor).

### Statistical analysis

GraphPad Prism 5.0 software (GraphPad-Prism Software Inc., San Diego, CA) was used for statistical analyses. Categorical variables were compared by χ^2^ test. Continuous variables were compared using independent two sample t-test. Univariate and multivariate analyses were performed by the Cox proportional hazard model. Survival curves were done by the Kaplan–Meier method (the log-rank test). All data are presented as Means ± standard deviations (SD). Pearson correlation analysis was used to evaluate the association between the expression of Apollon and Smac. The logistic regression analysis was used to assess the association between the expression of Apollon and chemotherapy response. All tests were two-tailed and a *P* < 0.05 was considered to be statistically significant.

### Study approval

The use of human tissue samples and clinical data was approved by the ethics committee of Fudan University and Nanchang University. All donors were informed of the aim of the study and gave consent to donate their samples. Animal experiments were performed according to the criteria outlined in the Guide for the Care and Use of Laboratory Animals, prepared by the National Academy of Sciences and published by the National Institutes of Health, and also approved by the ethics committee of Fudan University.

## SUPPLEMENTARY MATERIAL FIGURES AND TABLES


