# Supplementary material for: Apollon modulates chemosensitivity in human esophageal squamous cell carcinoma
Source: Oncotarget. 2014 Jul 31;5(16):7183–97. doi: 10.18632/oncotarget.2293 (PMC4196194; doi:10.18632/oncotarget.2293)
Supplement: Supplementary file 1 [file oncotarget-05-7183-s001.pdf]

Apollon modulates chemosensitivity in human esophageal squamous cell carcinoma

Supplementary Material

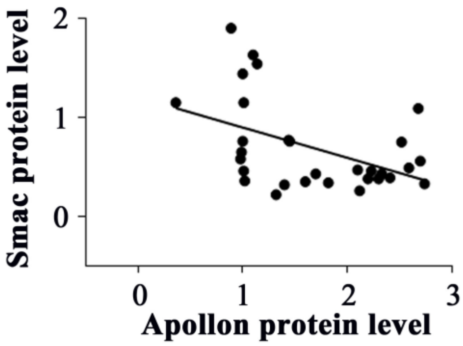

Supplementary Figure 1: Correlations between Apollon protein levels and Smac protein levels in 30 ESCC tissue samples.

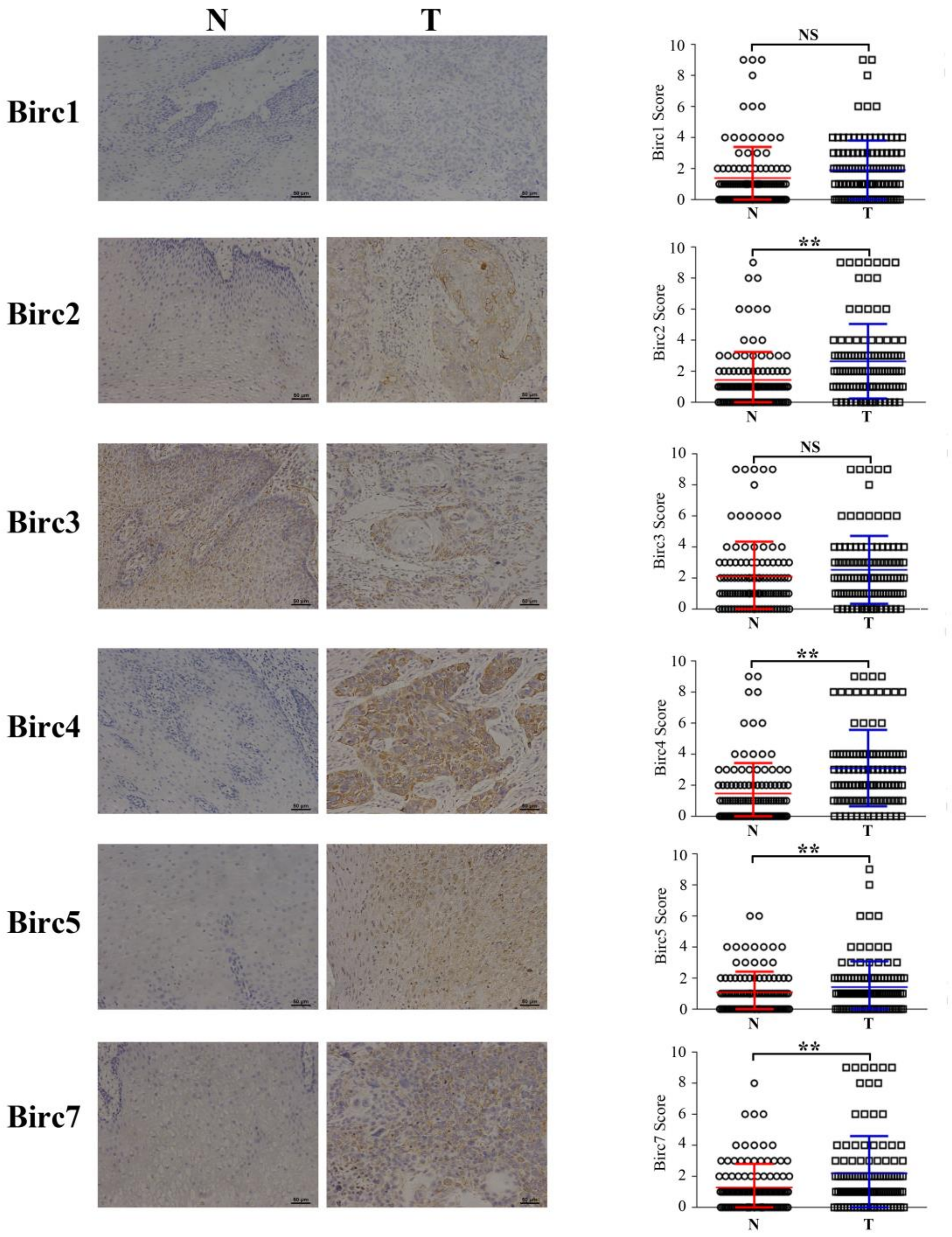

Supplementary Figure 2: Expression of the other members of inhibitor of apoptosis proteins (IAPs) in clinical ESCC samples. Left panel: Typical patterns of IAPs (Birc1, Birc2, Birc3, Birc4, Birc5, Birc7) staining in paired ESCC tissue samples. N, adjacent non-tumorous tissues; T, tumor tissues. Right panel: Scores of immunochemistry staining of IAPs in 111 ESCC patients. $**P < 0.01$.

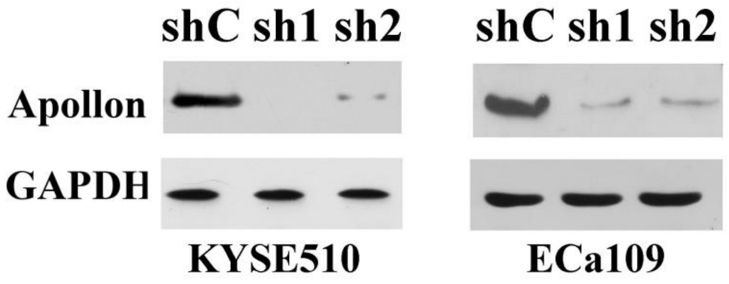

Supplementary Figure 3: Apollon knockdown efficiency in KYSE510 and Eca109 cells was testified by Western blotting.

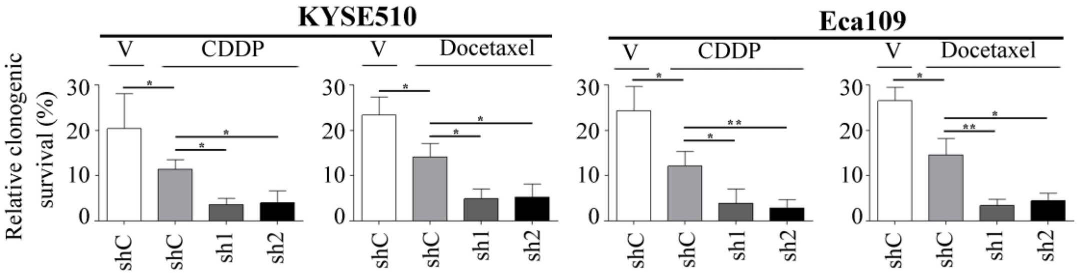

Supplementary Figure 4: Apollon knockdown enhanced cisplatin- or docetaxel-induced long-term cell growth inhibition. KYSE510 cells and Eca109 cells transfected with control shRNA (shC) or Apollon shRNAs (sh1, sh2) were treated with vehicle (V), CDDP (10 μM) or docetaxel (10 nM) for 6 hours, long-term cell viability was assessed by the colony formation assay and the relative survival was calculated. *$P < 0.05$ and **$P < 0.01$.

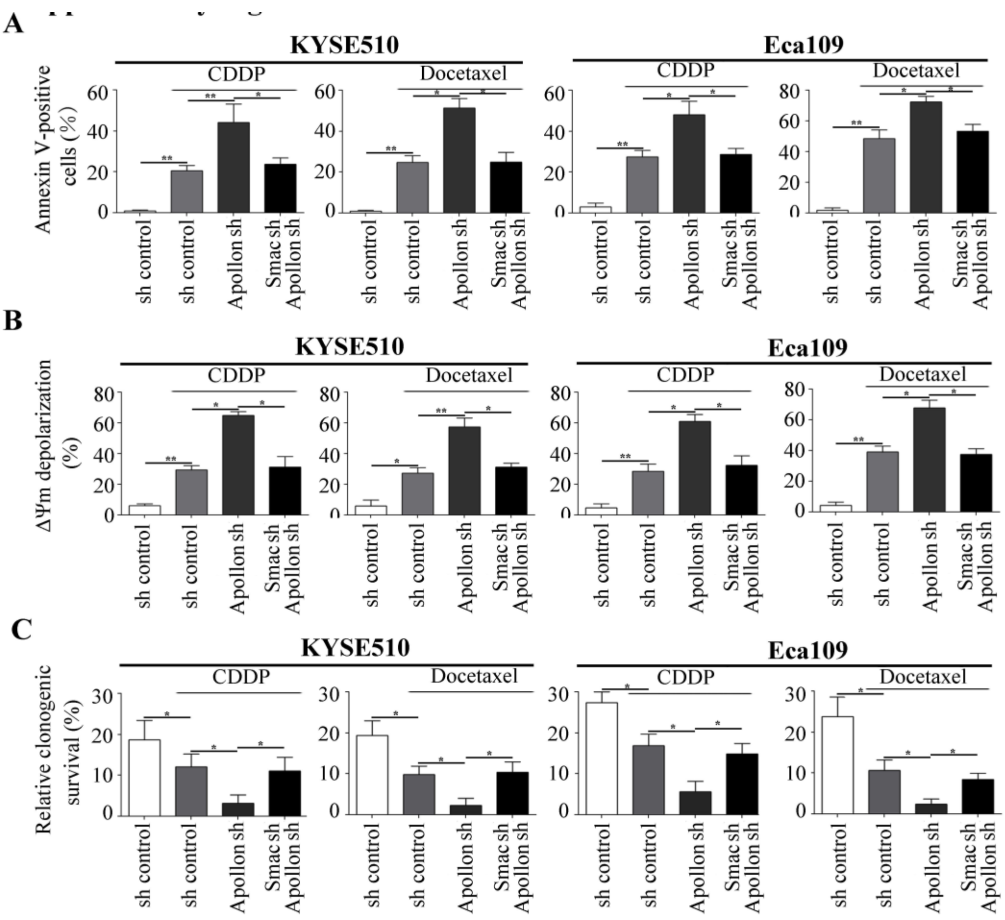

Supplementary Figure 5: Smac knockdown reversed the potentiated effects of Apollon knockdown on cisplatin (CDDP) or docetaxel-induced apoptotic events and long-term cell growth suppression in KYSE510 and Eca109 cells. Smac knockdown reversed the potentiated effect of Apollon knockdown on CDDP (10 μM) or docetaxel (10 nM) induced apoptosis (A), transmembrane potential (Δψm) depolarization (B) and long-term cell growth inhibition (C). **P* < 0.05 and ***P* < 0.01.

Supplementary Table 1: Clinical parameters of 111 ESCC patients

Patients	111
Age, years	
≤56	51
>56	60
Lymph nodes metastasis	
No	50
Yes	61
Distant metastasis	
No	6
Yes	105
Invasive depth	
Mucosa to muscularisproria	33
Adventitia to adjacent structure	78
Tumor size (cm)	
≤ 4	55
> 4	56
Position	
Upper and middle	90
Hypomere	21
TNM stage	
I, II	62
III, IV	49
Tumor differentiation	
I, II	89
III	22

Supplementary Table 2: Correlation between Apollon and clinicopathologic characteristics

	expression of Apollon		*P* values	OR (95%CI)
	Negative	Positive		
Patients	49	62		
Age (years)				
≤ 56	24	27		
> 56	25	35	0.569	1.244 (0.587-2.640)
Lymph nodes metastasis				
No	26	24		
Yes	23	38	0.133	1.790 (0.838-3.823)
Distant metastasis				
No	2	4		
Yes	47	58	0.587	0.617 (0.108-3.517)
Invasive depth				
Mucosa to muscularispropria	16	17		
Adventitia to adjacent structure	33	45	0.550	1.283 (0.567-2.906)
Tumor size (cm)				
≤ 4	26	29		
> 4	23	33	0.511	1.286 (0.607-2.725)
Position				
Upper and middle	40	50		
Hypomere	9	12	0.895	1.067 (0.409-2.783)
TNM stage				
I, II	35	27		
III, IV	20	29	0.103	1.880 (0.880-4.016)
Tumor differentiation				
I, II	39	50		
III	10	12	0.890	0.936 (0.366-2.391)

Supplementary Table 3: Clinical parameters of the chemotherapeutic response of 70 ESCC patients

Patients	70
Age, years	
≤56	22
>56	48
Lymph nodes metastasis	
No	32
Yes	38
Distant metastasis	
No	15
Yes	55
Invasive depth	
Mucosa to muscularisproria	24
Adventitia to adjacent structure	46
Tumor size (cm)	
≤ 4	27
> 4	43
Position	
Upper and middle	58
Hypomere	12
TNM stage	
I, II	32
III, IV	38
Tumor differentiation	
I, II	45
III	25
